# Supplementary material for: Multivariable two-sample Mendelian randomization estimates of the effects of intelligence and education on health
Source: eLife. 2019 Sep 17;8:e43990. doi: 10.7554/eLife.43990 (PMC6748790; doi:10.7554/eLife.43990)
Supplement: Supplementary file 1. [file elife-43990-supp1.docx]

**Intelligence, education and health, evidence from bidirectional two-sample Mendelian randomization**

**Supplementary data**

Neil M Davies*^1,2^, W. David Hill^3,4^, Emma L Anderson^1,2^, Eleanor Sanderson^1,2^, Ian J Deary^3,4^ and George Davey Smith^1,2^

^1^ Medical Research Council Integrative Epidemiology Unit, University of Bristol, BS8 2BN, United Kingdom.

^2^ Bristol Medical School, University of Bristol, Barley House, Oakfield Grove, Bristol, BS8 2BN, United Kingdom.

^3^ Centre for Cognitive Ageing and Cognitive Epidemiology, University of Edinburgh, Edinburgh, UK

^4^ Department of Psychology, University of Edinburgh, Edinburgh, UK

* Corresponding author (email:neil.davies@bristol.ac.uk, tel: +44 117 331 3417)

**Classification**: Social Sciences (Economic Sciences) and Biological Sciences (Genetics).

**Key Words**: Education, intelligence, UK Biobank, Mendelian randomization

**Supplementary File 1 -** **Figure 1**: Flow chart of inclusion and exclusion into the study sample. We restricted the samples to minimize sample overlap between the discovery samples (Hill et al. and Okbay et al.). Hill et al. used all UK Biobank participants who took the verbal-numeric reasoning tests. Therefore, the instrument-outcome associations for the two-sample analysis is restricted to the individuals who were not included in these analyses. The single sample analysis uses the Sniekers et al. intelligence GWAS. This did not include any samples from UK Biobank. Final sample sizes represent the maximum number of observations included in the analysis. Observations missing an outcome value were omitted from the analysis for each outcome.

**Single-sample exposure and outcome data from UKBB**

Took the verbal-numeric reasoning test and completed education question

N= 93,195

**Two-sample outcome data from UK BB.**

**Intelligence and education coefficients from Hill and Okbay et al.**

Did not take the verbal-numeric reasoning test

N= 138,670

In the interim release

N= 78,616

Not in the interim release

N= 203,064

Born in England

N=281,680

Not born in England N=53,291

Did not meet genotyping QC thresholds N=168,346

Related

Non-white or non-British

Did not attend study clinic N=8,780,136

Met genotyping QC thresholds N=334,971

Attended study clinic and consented N=503,317

Invited to participate in UK Biobank N = 9,283,453

**Supplementary File 1 - Figure 2**: Flow chart of inclusion and exclusion of SNPs into the analysis. First we restricted to the set of SNPs that were available in UK Biobank Haplotype Reference Consortium (HRC) panel, Hill et al. and Okbay et al. Next we clumped the GWAS results separately for intelligence and education to identify independent SNPs. Finally, we clumped a combined list of SNPs for both traits. This resulted in 219 SNPs, 181 and 75 associated with intelligence and education at p<5x10^-08^. There were 37 SNPs that associated with both intelligence and education at p<5x10^-08^ all SNPs were included in the analysis.

Clump combined list

(r^2^=0.01, distance=10,000Kb)

Total: 219 SNPs included in bivariate analysis

144 associated with intelligence

38 associated with education

37 associated with both

at p<5x10^-08^

Notes: Combined results clumped by selecting the SNP within a region most strongly associated with education in the Okbay GWAS (i.e. lowest p-value). Results were very similar when selecting the SNPs most strongly associated with intelligence in the Hill et al. intelligence GWAS. For the univariate analysis and the bidirectional education-intelligence results we clumped the intelligence and education GWAS separately using p-values from the respective GWAS. This results in 194 intelligence SNPs and 75 education SNPs, however, some of these SNPs represent common signals in the same genomic region.

Select SNPs associated with

intelligence at p<5x10^-08^

Clump GWAS

(r^2^=0.01, distance=10,000Kb)

197 SNPs

Common (7,303,122 SNPs)

Select SNPs associated with

education at p<5x10^-08^

Clump GWAS

(r^2^=0.01, distance=10,000Kb)

79 SNPs

Hill et al. (7,710,315 SNPs)

Okbay et al. (8,146,840 SNPs)

UK Biobank HRC (11,554,957 SNPs)

**Supplementary File 1 - Figure 3**: The total (univariable) effects of intelligence and education on the outcomes. Estimated using the 194 Hill et al. SNPs and 75 Okbay et al. SNPs. Estimated using **two sample multivariable Mendelian randomization**. The effects were estimated using inverse variance weighted summary data methods. For MR-Egger, weighted median and mode please see Supplementary Table 6.

Notes: Univariate analysis independently clumped using each GWAS. Hence 194 SNPs selected for intelligence. The results are unchanged if the 181 SNPs associated with intelligence in the bivariate analysis are used instead.

**Supplementary File 1 - Figure 4**: The direct effects of SD changes in intelligence and years of education on later outcomes in UK Biobank. **Single sample multivariable Mendelian randomization** using polygenic scores of the 16 intelligence SNPs identified the Sniekers et al. and 75 SNPs identified in the discovery sample of the Okbay et al. at p<5x10^-08^. Sniekers et al. only included the UK Biobank interim release, so the results below were estimated in an entirely independent sample excluding individuals in the interim release.

**Supplementary File 1 - Figure 5**: The direct effects of SD changes in intelligence and years of education on later outcomes in UK Biobank. **Single sample multivariable Mendelian randomization** using polygenic scores of the 16 intelligence SNPs identified the Sniekers et al. and 75 SNPs identified in the discovery sample of the Okbay et al. at p<5x10^-08^. Sniekers et al. only included the UK Biobank interim release, so the results below were estimated in an entirely independent sample excluding individuals in the interim release. Additionally adjusted for relative body size and height aged 8, birthweight, distance of birth from London, birth location index of multiple deprivation, birth location, having been breastfed, whether father or mother alive, and number of siblings. Sample size ranges from 30,487 to 33,741.

**Supplementary File 1 - Figure 6**: Bias component plot for intelligence against a set of potential baseline confounders.

**Supplementary File 1 - Figure 7**: Bias component plot for education attainment against a set of potential baseline confounders.

**Supplementary File 1 - Table 1**: The estimated effect of a unit change in intelligence on SD of years of schooling (194 SNPs). Summary estimates of the effect of each SNP on intelligence taken from Hill et al. and the effect of the SNPs on education taken from the UK Biobank sample excluding those who took the intelligence test and those in the interim sample. Estimated using **two sample multivariable Mendelian randomization**.

|  | Point | Standard | Confidence interval | |  |
| --- | --- | --- | --- | --- | --- |
|  | estimate | error | Lower | Upper | P-value |
| IVW | 0.523 | 0.020 | 0.483 | 0.562 | 2.16x10^-145^ |
| MR-Egger slope | 0.562 | 0.097 | 0.372 | 0.751 | 6.82x10^-09^ |
| MR-Egger intercept | -0.001 | 0.002 | -0.005 | 0.003 | 0.681 |
| Weighted median | 0.531 | 0.024 | 0.484 | 0.579 | 6.30x10^-107^ |
| Weighted mode | 0.516 | 0.081 | 0.358 | 0.674 | 1.52x10^-10^ |

Notes: adjusted for month of birth, year of birth, sex, interaction of sex and year of birth and the first 40 principal components. Q=368.7 (p<0.0001), I^2^_gx_=34.72%, I^2^=0.48 (95%CI: 0.38 to 0.56), H=1.4 (95%CI: 1.3 to 1.5). The 194 SNPs were selected by clumping on the intelligence GWAS (Hill et al.) and selecting SNPs associated at p<5x10^-08^. The results are very similar if the 181 SNPs used in the bivariate analysis are used instead.

**Supplementary File 1 - Table 2**: The estimated effect of an additional SD years of schooling on intelligence (75 SNPs). Summary estimates of the effect of each SNP on education taken from the discovery sample of Okbay et al. and the effect of the SNPs on intelligence taken from the UK Biobank sample restricted to those with who took the intelligence test and were not included in the interim sample. Estimated using **two sample multivariable Mendelian randomization**.

|  | Point | Standard | Confidence interval | |  |
| --- | --- | --- | --- | --- | --- |
|  | estimate | error | Lower | Upper | P-value |
| IVW | 0.766 | 0.046 | 0.676 | 0.857 | 2.96x10^-62^ |
| MR-Egger slope | 0.257 | 0.242 | -0.217 | 0.730 | 0.289 |
| MR-Egger intercept | 0.009 | 0.004 | 0.001 | 0.016 | 0.032 |
| Weighted median | 0.715 | 0.051 | 0.616 | 0.814 | 2.324x10^-45^ |
| Weighted mode | 0.560 | 0.119 | 0.327 | 0.792 | 2.33x10^-06^ |

Notes: adjusted for month of birth, year of birth, sex, interaction of sex and year of birth and the first 40 principal components. Q=183.3 (p<0.0001), I^2^_gx_ =12.54%, I^2^=0.60 (95%CI: 0.48 to 0.69), H=1.6 (95%CI: 1.4 to 1.8).

**Supplementary File 1 - Table 3**: **Two sample multivariable** estimates of the direct effects of education and intelligence on outcomes. Two sample Mendelian randomization using 181 Hill and 75 Okbay GWAS SNPs. Clumped using Okbay GWAS.

|  | Intelligence (Hill et al.) | | | | | Education (Okbay et al.) | | | | |
| --- | --- | --- | --- | --- | --- | --- | --- | --- | --- | --- |
|  | Point | Standard | Confidence interval | |  | Point | Point | Confidence interval | |  |
|  | estimate | error | Lower | Upper | P-value | estimate | error | Lower | Upper | P-value |
| Hypertension | -0.028 | 0.023 | -0.073 | 0.016 | 0.22 | -0.022 | 0.035 | -0.091 | 0.047 | 0.53 |
| Diabetes | -0.002 | 0.010 | -0.021 | 0.016 | 0.81 | -0.012 | 0.014 | -0.040 | 0.017 | 0.42 |
| Stroke | -0.006 | 0.004 | -0.014 | 0.002 | 0.17 | 0.003 | 0.006 | -0.010 | 0.015 | 0.65 |
| Heart attack | -0.011 | 0.006 | -0.022 | 0.001 | 0.07 | 0.000 | 0.009 | -0.018 | 0.018 | 0.98 |
| Episodes of depression | 0.000 | 0.022 | -0.044 | 0.044 | 1.00 | -0.024 | 0.034 | -0.091 | 0.043 | 0.48 |
| Cancer | -0.012 | 0.014 | -0.039 | 0.016 | 0.40 | 0.001 | 0.020 | -0.038 | 0.040 | 0.97 |
| Mortality | -0.006 | 0.005 | -0.016 | 0.004 | 0.26 | 0.004 | 0.008 | -0.011 | 0.020 | 0.59 |
| Ever smoker | -0.003 | 0.025 | -0.052 | 0.047 | 0.91 | -0.077 | 0.042 | -0.159 | 0.005 | 0.07 |
| Smoker | -0.017 | 0.013 | -0.043 | 0.009 | 0.21 | -0.031 | 0.020 | -0.070 | 0.008 | 0.12 |
| Income over £18k | 0.052 | 0.019 | 0.015 | 0.089 | 0.007 | 0.108 | 0.030 | 0.049 | 0.166 | 4.2x10^-04^ |
| Income over £31k | 0.046 | 0.022 | 0.002 | 0.089 | 0.04 | 0.166 | 0.034 | 0.099 | 0.232 | 2.1x10^-06^ |
| Income over £52k | 0.047 | 0.020 | 0.008 | 0.086 | 0.02 | 0.129 | 0.031 | 0.067 | 0.190 | 5.7x10^-05^ |
| Income over £100k | 0.008 | 0.008 | -0.009 | 0.024 | 0.37 | 0.050 | 0.013 | 0.024 | 0.076 | 2.1x10^-04^ |
| Grip strength (kg)* | 0.010 | 0.439 | -0.851 | 0.871 | 0.98 | 0.582 | 0.620 | -0.633 | 1.798 | 0.35 |
| Height (cm)* | 0.993 | 0.634 | -0.250 | 2.235 | 0.12 | 0.620 | 0.898 | -1.141 | 2.380 | 0.49 |
| BMI (kg/m2)* | -0.361 | 0.325 | -0.997 | 0.276 | 0.27 | -0.995 | 0.477 | -1.930 | -0.060 | 0.04 |
| Diastolic blood pressure (mmHg)* | -0.339 | 0.561 | -1.439 | 0.761 | 0.55 | -1.342 | 0.854 | -3.016 | 0.333 | 0.12 |
| Systolic blood pressure (mmHg)* | -1.422 | 0.932 | -3.248 | 0.405 | 0.13 | -0.880 | 1.416 | -3.655 | 1.896 | 0.54 |
| Alcohol consumption (1 low, 5 high)* | 0.190 | 0.067 | 0.059 | 0.320 | 0.005 | 0.212 | 0.102 | 0.011 | 0.412 | 0.04 |
| Hours of television viewing per day* | -0.060 | 0.072 | -0.202 | 0.081 | 0.40 | -0.778 | 0.112 | -0.997 | -0.560 | 3.6x10^-11^ |
| Vigorous physical activity (days/week)* | -0.343 | 0.075 | -0.491 | -0.196 | 8.1x10^-06^ | 0.312 | 0.114 | 0.088 | 0.536 | 0.007 |
| Moderate physical activity (days/week)* | -0.308 | 0.098 | -0.500 | -0.116 | 0.002 | -0.040 | 0.153 | -0.339 | 0.260 | 0.80 |

**Supplementary File 1 - Table 4**: **Single sample multivariable** estimates of the direct effects of intelligence and education on outcomes. Using allele scores of 16 Sniekers et al. and 75 Okbay et al. SNPs.

|  | Intelligence (Sniekers et al.) | | | | |  | Education (Okbay et al.) | | | | |  |
| --- | --- | --- | --- | --- | --- | --- | --- | --- | --- | --- | --- | --- |
|  | Point | Standard | Confidence interval | |  | Sanderson- | Point | Standard | Confidence interval | |  | Sanderson- |
|  | estimate | error | Lower | Upper | P-value | Windmeijer F | estimate | error | Lower | Upper | P-value | Windmeijer F |
| Hypertension | -0.013 | 0.075 | -0.160 | 0.133 | 0.33 | 25.7 | -0.087 | 0.089 | -0.262 | 0.088 | 0.33 | 25.7 |
| Diabetes | 0.003 | 0.034 | -0.063 | 0.069 | 0.42 | 26.5 | -0.032 | 0.039 | -0.109 | 0.045 | 0.42 | 26.5 |
| Stroke | 0.027 | 0.021 | -0.014 | 0.068 | 0.010 | 27.1 | -0.040 | 0.024 | -0.088 | 0.008 | 0.010 | 27.1 |
| Heart attack | 0.040 | 0.025 | -0.008 | 0.089 | 0.02 | 27.1 | -0.069 | 0.029 | -0.127 | -0.012 | 0.02 | 27.1 |
| Episodes of depression | 0.105 | 0.084 | -0.059 | 0.270 | 0.07 | 25.8 | -0.175 | 0.098 | -0.366 | 0.017 | 0.07 | 25.8 |
| Cancer | -0.068 | 0.057 | -0.180 | 0.044 | 0.23 | 27.2 | 0.080 | 0.067 | -0.051 | 0.210 | 0.23 | 27.2 |
| Mortality | -0.024 | 0.018 | -0.059 | 0.012 | 0.28 | 27.3 | 0.022 | 0.021 | -0.018 | 0.062 | 0.28 | 27.3 |
| Ever smoker | 0.126 | 0.086 | -0.044 | 0.295 | 0.02 | 28.8 | -0.233 | 0.100 | -0.429 | -0.037 | 0.02 | 28.8 |
| Smoker | 0.061 | 0.047 | -0.032 | 0.154 | 0.02 | 28.8 | -0.135 | 0.056 | -0.246 | -0.025 | 0.02 | 28.8 |
| Income over £18k | 0.063 | 0.064 | -0.063 | 0.188 | 0.01 | 21.6 | 0.202 | 0.079 | 0.047 | 0.356 | 0.01 | 21.6 |
| Income over £31k | 0.046 | 0.086 | -0.122 | 0.215 | 0.002 | 21.6 | 0.314 | 0.103 | 0.111 | 0.516 | 0.002 | 21.6 |
| Income over £52k | -0.041 | 0.091 | -0.218 | 0.137 | 0.001 | 21.6 | 0.364 | 0.113 | 0.143 | 0.585 | 0.001 | 21.6 |
| Income over £100k | -0.037 | 0.043 | -0.121 | 0.048 | 0.005 | 21.6 | 0.152 | 0.055 | 0.045 | 0.260 | 0.005 | 21.6 |
| Grip strength (kg)* | 0.040 | 1.106 | -2.127 | 2.207 | 0.79 | 26.3 | 0.340 | 1.258 | -2.126 | 2.806 | 0.78 | 26.3 |
| Height (cm)* | -1.467 | 1.188 | -3.796 | 0.862 | 0.01 | 26.5 | 3.522 | 1.418 | 0.743 | 6.301 | 0.01 | 26.5 |
| BMI (kg/m2)* | 1.235 | 0.931 | -0.590 | 3.060 | 3.9x10^-04^ | 25.8 | -3.899 | 1.100 | -6.055 | -1.743 | 3.9x10^-04^ | 25.8 |
| Diastolic blood pressure (mmHg)* | -0.883 | 1.606 | -4.030 | 2.264 | 0.20 | 29.3 | -2.406 | 1.861 | -6.053 | 1.241 | 0.19 | 29.3 |
| Systolic blood pressure (mmHg)* | -1.605 | 2.681 | -6.859 | 3.649 | 0.40 | 29.3 | -2.665 | 3.153 | -8.844 | 3.514 | 0.39 | 29.3 |
| Alcohol consumption (1 low, 5 high)* | -0.175 | 0.264 | -0.693 | 0.343 | 0.02 | 27.0 | 0.746 | 0.311 | 0.137 | 1.356 | 0.02 | 27.0 |
| Hours of television viewing per day* | 0.324 | 0.317 | -0.298 | 0.945 | 1.0x10^-05^ | 28.3 | -1.617 | 0.366 | -2.335 | -0.899 | 1.0x10^-05^ | 28.3 |
| Vigorous physical activity (days/week)* | -1.051 | 0.364 | -1.764 | -0.338 | 0.02 | 25.8 | 1.003 | 0.421 | 0.178 | 1.828 | 0.02 | 25.8 |
| Moderate physical activity (days/week)* | -1.425 | 0.478 | -2.361 | -0.489 | 0.02 | 24.7 | 1.312 | 0.561 | 0.211 | 2.412 | 0.02 | 24.7 |

**Supplementary File 1- Table 5**: **Single sample multivariable** estimates of the direct effects of education and intelligence on outcomes. Using allele scores of 16 Sniekers and 486 Lee SNPs . There is sample overlap between the outcomes and the discovery sample for the GWAS.

|  | Education (Lee et al.) | | | | |  | Intelligence (Sniekers et al.) | | | | |  |
| --- | --- | --- | --- | --- | --- | --- | --- | --- | --- | --- | --- | --- |
|  | Point | Standard | Confidence interval | |  | Sanderson- | Point | Standard | Confidence interval | |  | Sanderson- |
|  | estimate | error | Lower | Upper | P-value | Windmeijer F | estimate | error | Lower | Upper | P-value | Windmeijer F |
| Hypertension | -0.066 | 0.034 | -0.132 | 0.000 | 0.05 | 129.6 | -0.012 | 0.035 | -0.082 | 0.057 | 0.73 | 126.7 |
| Diabetes | -0.027 | 0.016 | -0.058 | 0.004 | 0.09 | 133.4 | 0.000 | 0.017 | -0.033 | 0.033 | 0.99 | 130.5 |
| Stroke | -0.023 | 0.009 | -0.041 | -0.005 | 0.01 | 133.1 | 0.014 | 0.010 | -0.005 | 0.033 | 0.16 | 130.0 |
| Heart attack | -0.014 | 0.010 | -0.034 | 0.006 | 0.16 | 133.1 | 0.001 | 0.011 | -0.020 | 0.022 | 0.93 | 130.0 |
| Episodes of depression | -0.102 | 0.037 | -0.175 | -0.029 | 0.006 | 129.6 | 0.040 | 0.039 | -0.037 | 0.117 | 0.31 | 126.7 |
| Cancer | 0.020 | 0.026 | -0.032 | 0.072 | 0.45 | 132.5 | -0.021 | 0.027 | -0.074 | 0.033 | 0.44 | 129.2 |
| Mortality | -0.005 | 0.007 | -0.019 | 0.010 | 0.51 | 134.1 | -0.001 | 0.008 | -0.016 | 0.015 | 0.94 | 131.0 |
| Ever smoker | -0.198 | 0.041 | -0.277 | -0.118 | 1.1x10^-06^ | 136.4 | 0.090 | 0.043 | 0.006 | 0.174 | 0.04 | 133.0 |
| Smoker | -0.118 | 0.023 | -0.163 | -0.073 | 3.1x10^-07^ | 136.4 | 0.047 | 0.024 | -0.001 | 0.095 | 0.05 | 133.0 |
| Income over £18k | 0.138 | 0.030 | 0.079 | 0.197 | 5.0x10^-06^ | 119.7 | 0.071 | 0.032 | 0.008 | 0.133 | 0.03 | 117.1 |
| Income over £31k | 0.165 | 0.035 | 0.097 | 0.234 | 2.3x10^-06^ | 119.7 | 0.099 | 0.038 | 0.025 | 0.173 | 0.009 | 117.1 |
| Income over £52k | 0.192 | 0.033 | 0.128 | 0.257 | 4.3x10^-09^ | 119.7 | 0.023 | 0.035 | -0.045 | 0.092 | 0.50 | 117.1 |
| Income over £100k | 0.073 | 0.018 | 0.038 | 0.109 | 4.8x10^-05^ | 119.7 | 0.002 | 0.019 | -0.034 | 0.038 | 0.91 | 117.1 |
| Grip strength (kg)* | 0.675 | 0.525 | -0.353 | 1.703 | 0.20 | 134.8 | -0.254 | 0.555 | -1.343 | 0.834 | 0.65 | 131.9 |
| Height (cm)* | 2.477 | 0.533 | 1.432 | 3.522 | 3.3x10^-06^ | 134.5 | -0.649 | 0.555 | -1.736 | 0.439 | 0.24 | 131.7 |
| BMI (kg/m2)* | -1.963 | 0.371 | -2.691 | -1.235 | 1.2x10^-07^ | 133.1 | 0.265 | 0.402 | -0.522 | 1.052 | 0.51 | 130.3 |
| Diastolic blood pressure (mmHg)* | -1.098 | 0.753 | -2.574 | 0.378 | 0.15 | 138.5 | -0.915 | 0.794 | -2.471 | 0.642 | 0.25 | 135.1 |
| Systolic blood pressure (mmHg)* | -1.354 | 1.316 | -3.932 | 1.224 | 0.30 | 138.5 | -1.940 | 1.395 | -4.674 | 0.795 | 0.16 | 135.2 |
| Alcohol consumption (1 low, 5 high)* | 0.525 | 0.115 | 0.300 | 0.750 | 4.7x10^-06^ | 133.0 | -0.031 | 0.121 | -0.268 | 0.207 | 0.80 | 129.7 |
| Hours of television viewing per day* | -1.073 | 0.145 | -1.358 | -0.788 | 1.5x10^-13^ | 128.8 | 0.076 | 0.154 | -0.225 | 0.378 | 0.62 | 125.7 |
| Vigorous physical activity (days/week)* | 0.388 | 0.164 | 0.067 | 0.708 | 0.02 | 125.7 | -0.631 | 0.171 | -0.967 | -0.295 | 2.3x10^-04^ | 123.6 |
| Moderate physical activity (days/week)* | 0.268 | 0.190 | -0.106 | 0.641 | 0.16 | 129.0 | -0.707 | 0.201 | -1.101 | -0.312 | 4.4x10^-04^ | 126.9 |

**Supplementary File 1 - Table 6**: Univariate effects of cognition and education using two-sample Mendelian randomization (“IVW – Univariate”) and using a within family model using siblings in the UK Biobank.

|  |  | Education (Okbay et al) | | | | | | Cognition (Sniekers et al) | | | | |
| --- | --- | --- | --- | --- | --- | --- | --- | --- | --- | --- | --- | --- |
|  |  | Point | Standard | Confidence interval | |  | Point | | Standard | Confidence interval | |  |
|  |  | estimate | error | Lower | Upper | P-value | estimate | | error | Lower | Upper | P-value |
| Hypertension | IVW - univariate | -0.045 | 0.018 | -0.080 | -0.009 | 0.01 | -0.037 | | 0.009 | -0.055 | -0.020 | 2.8x10^-05^ |
| Hypertension | Within families | 0.192 | 0.147 | -0.095 | 0.480 | 0.19 | 0.635 | | 0.983 | -1.292 | 2.562 | 0.52 |
| Diabetes | IVW - univariate | -0.014 | 0.007 | -0.029 | 0.000 | 0.05 | -0.009 | | 0.004 | -0.016 | -0.001 | 0.03 |
| Diabetes | Within families | -0.015 | 0.067 | -0.147 | 0.117 | 0.83 | -0.141 | | 0.303 | -0.736 | 0.453 | 0.64 |
| Stroke | IVW - univariate | -0.003 | 0.004 | -0.010 | 0.004 | 0.38 | -0.004 | | 0.002 | -0.008 | -0.001 | 0.02 |
| Stroke | Within families | 0.003 | 0.041 | -0.078 | 0.084 | 0.94 | 0.053 | | 0.149 | -0.240 | 0.346 | 0.72 |
| Heart attack | IVW - univariate | -0.013 | 0.005 | -0.022 | -0.003 | 0.01 | -0.011 | | 0.002 | -0.016 | -0.007 | 1.1x10^-06^ |
| Heart attack | Within families | -0.020 | 0.054 | -0.126 | 0.086 | 0.71 | -0.126 | | 0.214 | -0.546 | 0.294 | 0.56 |
| Episodes of depression | IVW - univariate | -0.016 | 0.017 | -0.050 | 0.018 | 0.35 | -0.009 | | 0.009 | -0.027 | 0.008 | 0.3 |
| Episodes of depression | Within families | 0.139 | 0.178 | -0.209 | 0.487 | 0.44 | 0.882 | | 1.088 | -1.250 | 3.014 | 0.42 |
| Cancer | IVW - univariate | -0.012 | 0.011 | -0.033 | 0.009 | 0.27 | -0.010 | | 0.006 | -0.021 | 0.002 | 0.1 |
| Cancer | Within families | -0.304 | 0.164 | -0.625 | 0.016 | 0.06 | -0.533 | | 0.659 | -1.825 | 0.759 | 0.42 |
| Mortality | IVW - univariate | 0.001 | 0.004 | -0.008 | 0.010 | 0.83 | -0.002 | | 0.002 | -0.006 | 0.002 | 0.33 |
| Mortality | Within families | 0.014 | 0.043 | -0.069 | 0.098 | 0.74 | 0.091 | | 0.146 | -0.195 | 0.377 | 0.53 |
| Ever smoker | IVW - univariate | -0.077 | 0.025 | -0.125 | -0.028 | 0.002 | -0.046 | | 0.010 | -0.067 | -0.026 | 1.0x10^-05^ |
| Ever smoker | Within families | -0.256 | 0.190 | -0.629 | 0.117 | 0.18 | -0.083 | | 0.458 | -0.980 | 0.814 | 0.86 |
| Smoker | IVW - univariate | -0.045 | 0.011 | -0.066 | -0.023 | 4.3x10^-05^ | -0.034 | | 0.006 | -0.045 | -0.023 | 7.9x10^-10^ |
| Smoker | Within families | -0.282 | 0.136 | -0.550 | -0.015 | 0.04 | -0.197 | | 0.323 | -0.831 | 0.437 | 0.54 |
| Income over £18k | IVW - univariate | 0.157 | 0.015 | 0.126 | 0.187 | 3.5x10^-24^ | 0.108 | | 0.008 | 0.092 | 0.123 | 1.7x10^-43^ |
| Income over £18k | Within families | -0.147 | 0.317 | -0.769 | 0.474 | 0.64 | 0.705 | | 2.042 | -3.297 | 4.707 | 0.73 |
| Income over £31k | IVW - univariate | 0.225 | 0.018 | 0.189 | 0.261 | 9.4x10^-35^ | 0.127 | | 0.009 | 0.109 | 0.145 | 3.4x10^-43^ |
| Income over £31k | Within families | 0.351 | 0.364 | -0.362 | 1.064 | 0.33 | 4.305 | | 10.864 | -16.988 | 25.597 | 0.69 |
| Income over £52k | IVW - univariate | 0.191 | 0.017 | 0.159 | 0.224 | 5.3x10^-31^ | 0.112 | | 0.008 | 0.097 | 0.127 | 4.7x10^-47^ |
| Income over £52k | Within families | 0.608 | 0.443 | -0.260 | 1.476 | 0.17 | 3.641 | | 9.158 | -14.308 | 21.591 | 0.69 |
| Income over £100k | IVW - univariate | 0.062 | 0.007 | 0.048 | 0.077 | 9.5x10^-17^ | 0.034 | | 0.004 | 0.027 | 0.042 | 4.5x10^-19^ |
| Income over £100k | Within families | 0.178 | 0.190 | -0.195 | 0.552 | 0.35 | 0.269 | | 0.897 | -1.490 | 2.028 | 0.76 |
| Grip strength (kg)* | IVW - univariate | 0.594 | 0.340 | -0.073 | 1.261 | 0.08 | 0.270 | | 0.174 | -0.071 | 0.610 | 0.12 |
| Grip strength (kg)* | Within families | 3.654 | 2.537 | -1.319 | 8.627 | 0.15 | 6.364 | | 9.229 | -11.725 | 24.453 | 0.49 |
| Height (cm)* | IVW - univariate | 1.461 | 0.453 | 0.574 | 2.348 | 0.001 | 1.384 | | 0.251 | 0.892 | 1.876 | 3.5x10^-08^ |
| Height (cm)* | Within families | 0.000 | 1.634 | -3.203 | 3.202 | 1.00 | -3.646 | | 5.595 | -14.612 | 7.321 | 0.51 |
| BMI (kg/m2)* | IVW - univariate | -1.644 | 0.243 | -2.119 | -1.168 | 1.3x10^-11^ | -0.845 | | 0.132 | -1.104 | -0.587 | 1.5x10^-10^ |
| BMI (kg/m2)* | Within families | -2.107 | 1.548 | -5.141 | 0.927 | 0.17 | 1.543 | | 4.805 | -7.874 | 10.960 | 0.75 |
| Diastolic blood pressure (mmHg)* | IVW - univariate | -1.811 | 0.408 | -2.610 | -1.012 | 8.9x10^-06^ | -1.012 | | 0.213 | -1.430 | -0.594 | 2.1x10^-06^ |
| Diastolic blood pressure (mmHg)* | Within families | -1.977 | 4.006 | -9.829 | 5.876 | 0.62 | -5.574 | | 12.349 | -29.778 | 18.631 | 0.65 |
| Systolic blood pressure (mmHg)* | IVW - univariate | -2.516 | 0.696 | -3.880 | -1.152 | 3.0x10^-04^ | -1.725 | | 0.362 | -2.433 | -1.016 | 1.8x10^-06^ |
| Systolic blood pressure (mmHg)* | Within families | -0.411 | 7.000 | -14.130 | 13.308 | 0.95 | -24.449 | | 30.985 | -85.179 | 36.282 | 0.43 |
| Alcohol consumption (1 low, 5 high)* | IVW - univariate | 0.444 | 0.065 | 0.317 | 0.572 | 7.2x10^-12^ | 0.275 | | 0.030 | 0.217 | 0.334 | 3.3x10^-20^ |
| Alcohol consumption (1 low, 5 high)* | Within families | 0.399 | 0.476 | -0.533 | 1.332 | 0.4 | 0.617 | | 1.762 | -2.836 | 4.070 | 0.73 |
| Hours of television viewing per day* | IVW - univariate | -0.887 | 0.069 | -1.022 | -0.751 | 9.0x10^-38^ | -0.466 | | 0.035 | -0.534 | -0.397 | 2.8x10^-40^ |
| Hours of television viewing per day* | Within families | -0.823 | 0.520 | -1.843 | 0.196 | 0.11 | 0.988 | | 2.149 | -3.224 | 5.199 | 0.65 |
| Vigorous exercise (days/week)* | IVW - univariate | -0.014 | 0.067 | -0.145 | 0.116 | 0.83 | -0.178 | | 0.035 | -0.246 | -0.109 | 3.7x10^-07^ |
| Vigorous exercise (days/week)* | Within families | -2.406 | 1.137 | -4.635 | -0.176 | 0.03 | -1.520 | | 2.321 | -6.070 | 3.030 | 0.51 |
| Moderate exercise (days/week)* | IVW - univariate | -0.324 | 0.085 | -0.490 | -0.158 | 1.3x10^-04^ | -0.314 | | 0.041 | -0.395 | -0.234 | 2.3x10^-14^ |
| Moderate exercise (days/week)* | Within families | -0.867 | 0.915 | -2.661 | 0.926 | 0.34 | -0.396 | | 2.243 | -4.793 | 4.001 | 0.86 |
